# Supplementary material for: A proteogenomic atlas of the human neural retina
Source: Front Genet. 2024 Sep 19;15:1451024. doi: 10.3389/fgene.2024.1451024 (PMC11450717; doi:10.3389/fgene.2024.1451024)
Supplement: Supplementary file 3 [file DataSheet5.docx]

Supplementary Material

## Supplementary Figures and Tables

## Supplementary Figures

##
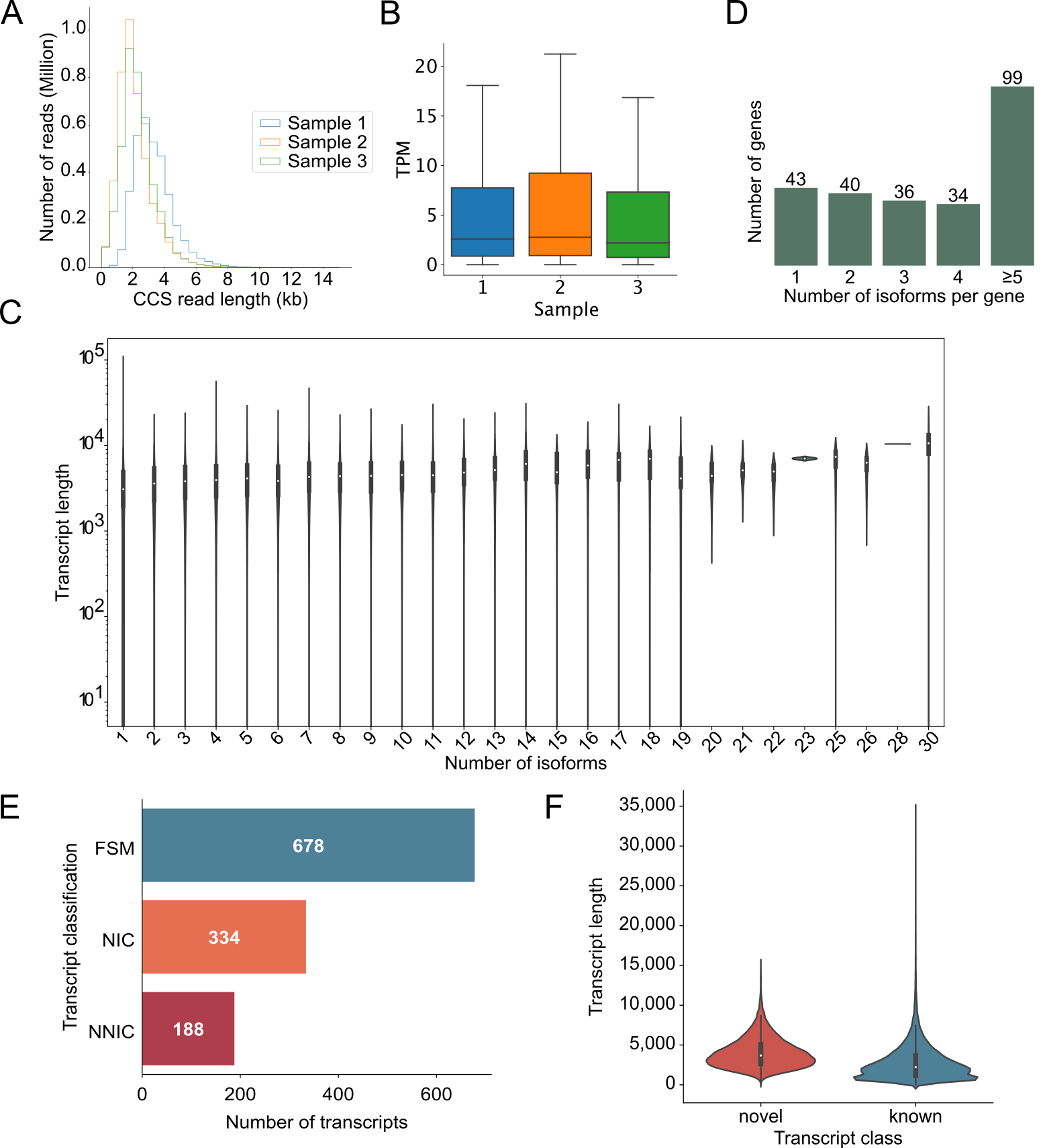


**Supplementary Figure 1: Additional information about the human neural retina transcriptome generated with PacBio long-read sequencing.** (A) Histogram (bin width = 500 bp) of the circular consensus sequence (CCS) read length in retina sample 1 (blue), retina sample 2 (orange), and retina sample 3 (green). (B) Violin plot showing the gene length depending on the number of isoforms detected for the gene. The Spearman correlation coefficient is 0.19 with a p-value < 0.05. (C) Comparison of normalized count distribution (in transcripts per million (TPM)) for sample 1 (blue), sample 2 (orange), and sample 3 (green). (D) Number of isoforms detected per RetNet gene across the three samples. (E) Number of RetNet transcripts from the three retina samples associated with each transcript class. The different classes are Full Splice Match (FSs) (blue), Novel In Catalog (NIC) (orange), and Novel Not In Catalog (NNIC) (red). (F) Comparison of the transcripts length between novel transcripts (red) and known transcripts (blue).


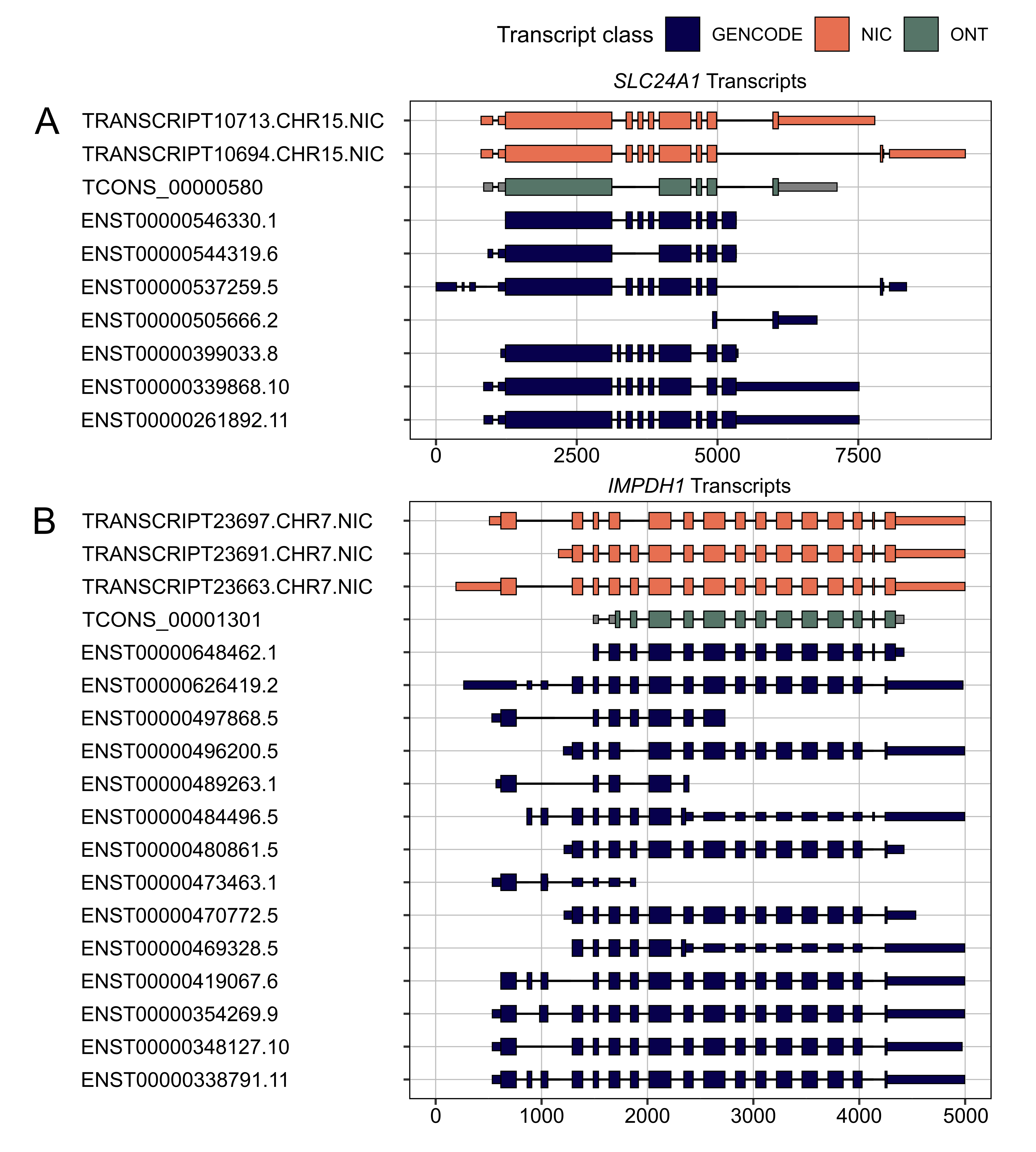


**Supplementary Figure 2. RetNet genes demonstrating the highest expression of a novel isoform.** PacBio transcripts are shown in orange, Oxford Nanopore Technology (ONT) sequencing StringTie2 transcripts in green, and reference GENCODE v39 transcripts in blue for **(A)** *SLC24A1* and **(B)** *IMPDH1*. We only show PacBio transcripts that result in a novel open reading frame and StringTie2 ONT transcripts that support novel PacBio transcripts. For all transcripts, the 5'-end is shown on the left and the 3'-end on the right.


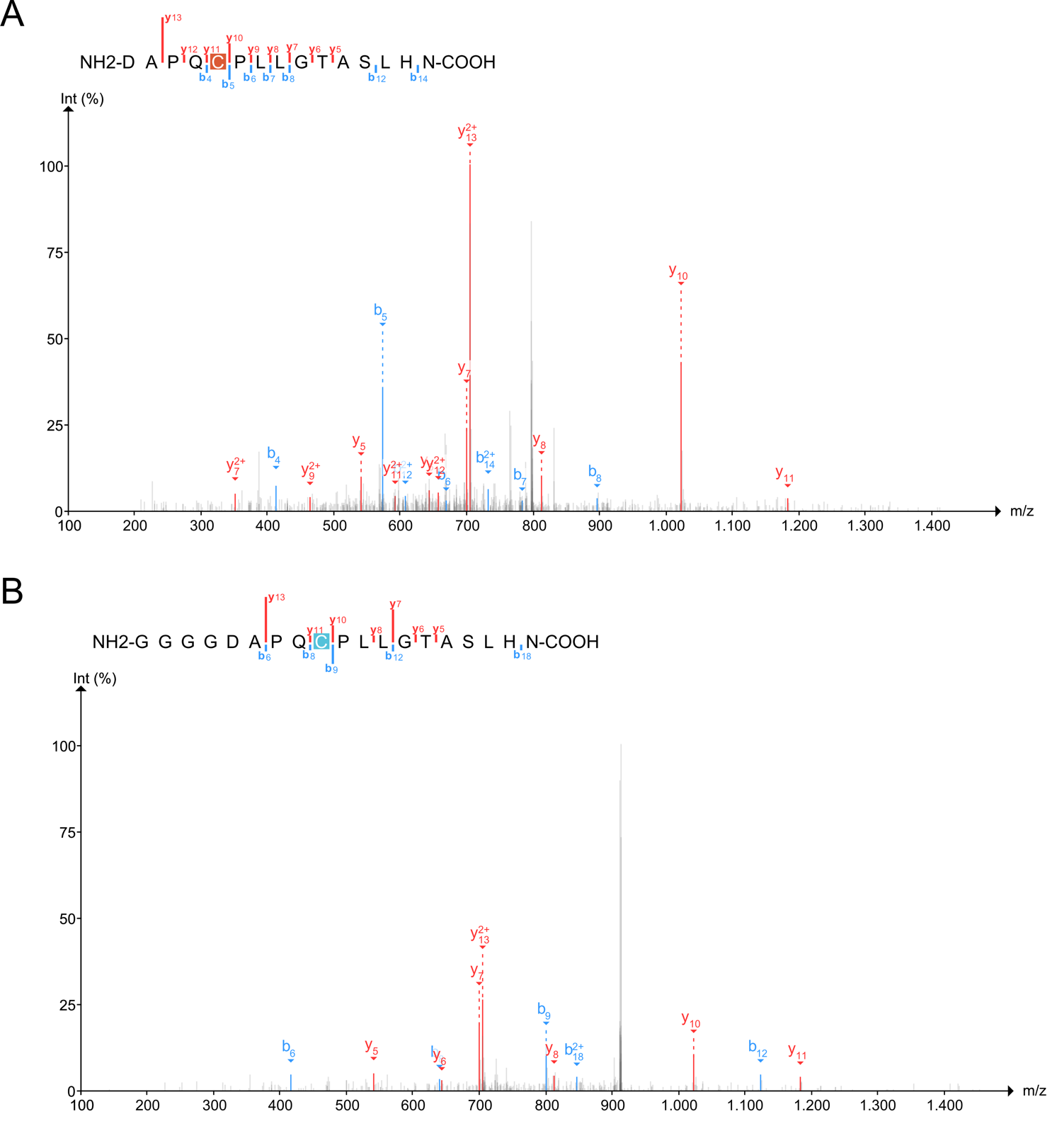
**Supplementary Figure 3: Spectra of peptides that confirm the alternative terminal exon in *IMPDH1*.** B-ions are shown in blue and y-ions in red.


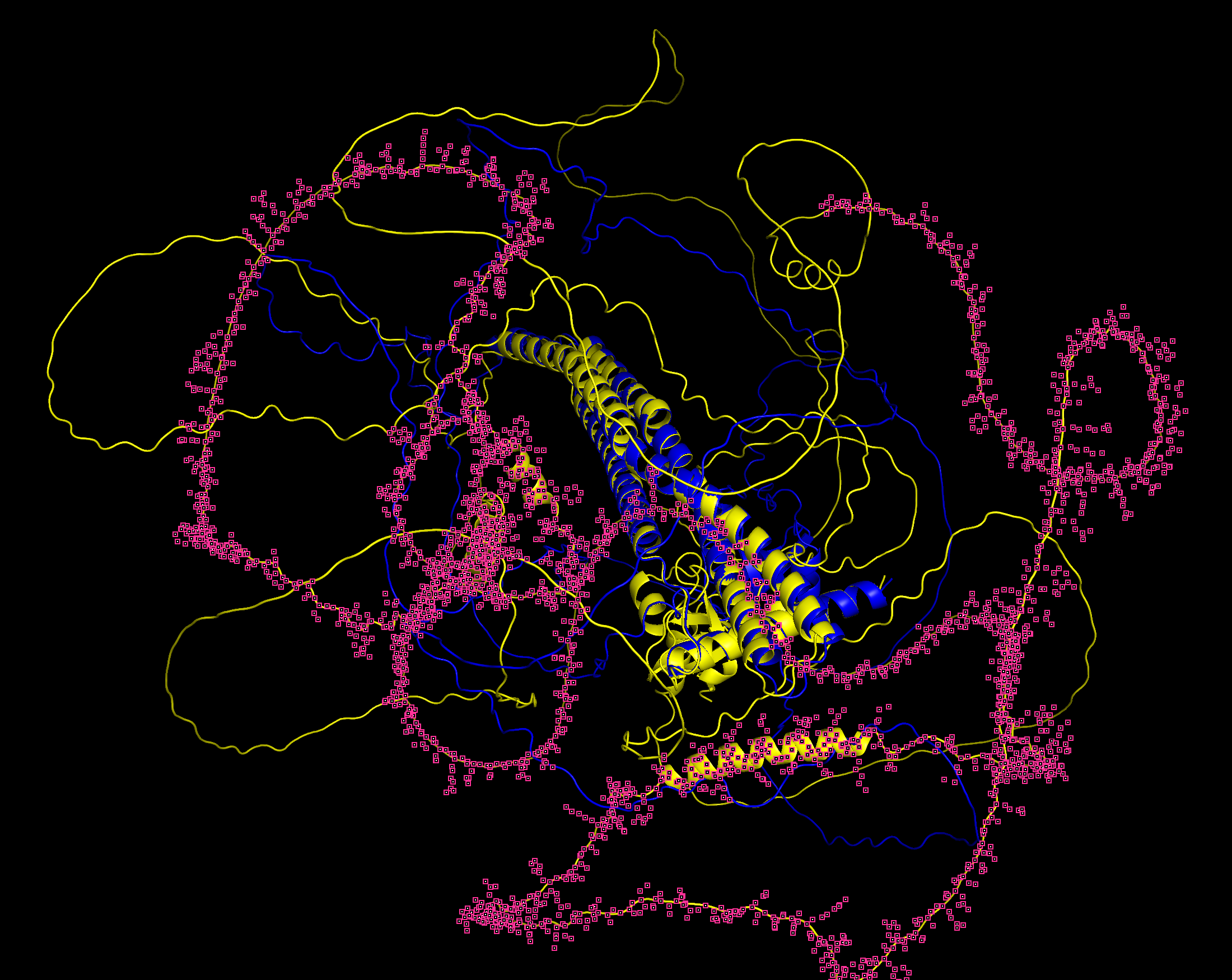


## Supplementary Figure 4: Visualization of AMPH AlphaFold predictions. AlphaFold predictions are shown for the UniProt protein H0Y7T8 (blue) and the novel open reading frame predicted for TRANSCRIPT6109.CHR7.NIC (yellow). The exon extension in the novel open reading frame is highlighted in pink.

## Supplementary Tables

**Supplementary** **Table 1: Description of the samples used for the study.**

| Sample ID | Sex | Age | Time until enucleation | RIN value |
| --- | --- | --- | --- | --- |
| Sample 1 | Male | 59 | 2 hours 35 minutes | 8.2 |
| Sample 2 | Male | 63 | 9 hours 5 minutes | 7.3 |
| Sample 3 | Female | 58 | 11 hours 55 minutes | 7.5 |

**Supplementary** **Table 2: Description of the samples used for Oxford Nanopore Technology sequencing.**

|  | Time until enucleation | RIN value | Reads (passed) |
| --- | --- | --- | --- |
| Sample 1 | < 20 hours | > 8 | 10,206,706 |
| Sample 2 | < 20 hours | > 8 | 18,463,865 |
| Sample 3 | < 20 hours | > 8 | 11,388,116 |

**Supplementary** **Table 3: Overview of the number of reads for each sample after the different analysis steps.** CCS = circular consensus sequence, FL –=full length, FLNC = full length non concatemer

|  | Sample 1 | Sample 2 | Sample 3 |
| --- | --- | --- | --- |
| Reads | 5,063,614 | 7,011,790 | 6,942,704 |
| CSS reads | 3,353,662 | 4,216,550 | 4,044,519 |
| FL reads | 2,956,840 | 3,646,936 | 3,541,082 |
| FLNC reads | 2,949,506 | 3,627,727 | 3,531,995 |
| FLNC with Poly(A) reads | 2,944,441 | 3,616,413 | 3,521,382 |

**Supplementary** **Table 4: Comparison of the transcript and protein classification of novel isoforms.** NIC = Novel In Catalog, NNIC = Novel Not In Catalog, pFSM = protein Full Splice Match, pNIC = protein Novel In Catalog, pNNIC = protein Novel Not In Catalog

| Transcript Classification | Protein Classification | Count |
| --- | --- | --- |
| NIC | pFSM | 1883 |
| NIC | pNIC | 1521 |
| NIC | PNNIC | 4526 |
| NNIC | pFSM | 1368 |
| NNIC | pNIC | 565 |
| NNIC | pNNIC | 2636 |

**Supplementary** **Table 5: Comparison of the number of transcripts and peptides identified with IsoQuant, SQANTI3, and TALON.**

|  | IsoQuant | SQANTI3 | TALON |
| --- | --- | --- | --- |
| Number of full-length isoforms | 58,541 | 353,381 | 188,346 |
| Number of novel isoforms | 22,138 | 130,796 | 51,477 |
| Number of novel ORFs | 12,499 | 20,330 | 24,892 |
| Number of peptides | 33,503 | 33,539 | 33,450 |
| Number of novel peptides | 12 | 49 | 66 |
