# Supplementary material for: A proteogenomic atlas of the human neural retina
Source: Front Genet. 2024 Sep 19;15:1451024. doi: 10.3389/fgene.2024.1451024 (PMC11450717; doi:10.3389/fgene.2024.1451024)
Supplement: Supplementary file 6 [file Image1.pdf]

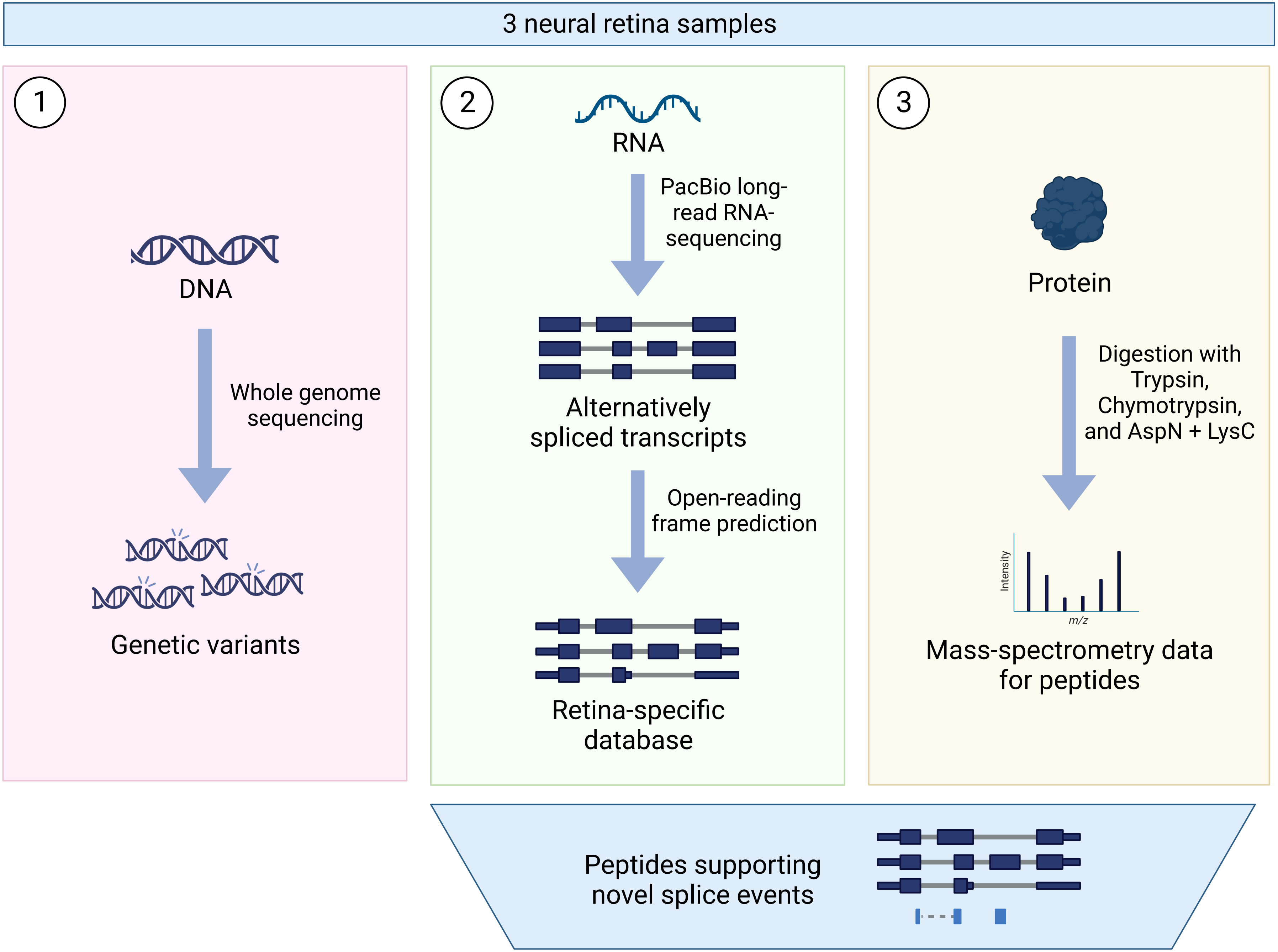
3 neural retina samples
1
DNA
Whole genome sequencing
Genetic variants
2
RNA
PacBio long-read RNA-sequencing
Alternatively spliced transcripts
Open-reading frame prediction
Retina-specific database
3
Protein
Digestion with Trypsin, Chymotrypsin, and AspN + LysC
Intensity
m/z
Mass-spectrometry data for peptides
Peptides supporting novel splice events
